# On the nucleation and initial film growth of rod-like organic molecules

**DOI:** 10.1016/j.susc.2016.02.015

**Published:** 2016-02-27

**Authors:** Adolf Winkler

**Affiliations:** Institute of Solid State Physics, Graz University of Technology, Petersgasse 16, A-8010 Graz, Austria

**Keywords:** Organic molecules, Thin films, Nucleation, Critical island size

## Abstract

In this article, some fundamental topics related to the initial steps of organic film growth are reviewed. General conclusions will be drawn based on experimental results obtained for the film formation of oligophenylene and pentacene molecules on gold and mica substrates. Thin films were prepared via physical vapor deposition under ultrahigh-vacuum conditions and characterized in-situ mainly by thermal desorption spectroscopy, and ex-situ by X-ray diffraction and atomic force microscopy. In this short review article the following topics will be discussed: What are the necessary conditions to form island-like films which are either composed of flat-lying or of standing molecules? Does a wetting layer exist below and in between the islands? What is the reason behind the occasionally observed bimodal island size distribution? Can one describe the nucleation process with the diffusion-limited aggregation model? Do the impinging molecules directly adsorb on the surface or rather via a hot-precursor state? Finally, it will be described how the critical island size can be determined by an independent measurement of the deposition rate dependence of the island density and the capture-zone distribution via a universal relationship.

## 1 Introduction

Thin films of organic semiconductors have attracted considerable interest in the recent past due to their promising applications in organic electronics. Various organic electronic devices are already on the market, e.g. solar cells, light emitting diodes, displays and radio frequency identification tags, to name just a few. The advantages of organic electronics in comparison to silicon-based technology are manifold. Properties such as low-cost of fabrication, material flexibility, large-scale fabrication by roll-to-roll printing at moderate temperatures and even possibly biocompatibility and biodegradability of the electronic material are scientifically and technologically attractive. Although for many purposes polymers are used, the application of small organic semiconducting molecules becomes increasingly important. In this case, organic thin films are mainly fabricated by physical vapor deposition. While this method weakens some of the above-mentioned advantages, it allows a very precise fabrication and tailoring of the organic films, which is an inevitable prerequisite for detailed investigations of the growth mechanism. However, despite those inherent advantages of physical vapor deposition techniques, many of the fundamental physical processes taking place during adsorption and organic thin film formation are still poorly understood.

The aim of this short review article is to summarize some of the most recent findings regarding the nucleation and growth of films composed of the rod-like organic molecules pentacene (C_22_H_14_), p-quaterphenyl (C_24_H_18_) and p-hexaphenyl (C_36_H_26_) on gold and mica substrates. These molecules are commonly used and increasingly relevant for applications in organic electronics. Pentacene (5A) is the most frequently used material to fabricate organic transistors, due to its high charge carrier mobility [1], while p-hexaphenyl (6P) was one of the first materials used to build blue light emitting diodes [2]. In the author’s view, the growth characteristics of these rod-like molecules can be seen as prime examples of organic film growth in general.

The organic molecules were deposited on the substrates in an ultra-high vacuum chamber by physical vapor deposition from a stainless steel Knudsen cell; the deposited amount was quantitatively measured with a quartz microbalance. The substrates were clipped onto a heatable sample holder which allowed the application of thermal desorption spectroscopy (TDS) to characterize the kinetics and energetics of adsorption and film formation. Additionally, Auger electron spectroscopy (AES) and X-ray photoelectron spectroscopy (XPS) were applied to chemically characterize the substrates and/or the thin films. Low-energy electron diffraction (LEED) was performed to study the structure of the monolayer films, while the structure and morphology of thick films were measured ex-situ by X-ray diffraction (XRD) and atomic force microscopy (AFM), respectively. Details on the experimental procedures can be found in a number of previous publications [3,4,5].

In this article, some frequently discussed issues in the context of the nucleation and growth of films composed of rod-like organic molecules will be addressed: Which parameters define whether the island-like film is composed of lying or upright standing molecules? Is there a wetting layer between and underneath the islands? Can the nucleation and growth be described by the classical diffusion-limited aggregation model? The discussion of these issues is mainly based on experimental work performed in my group at the Graz University of Technology and on theoretical work carried out by Alberto Pimpinelli at the Rice University, Texas.

## 2 Organic films composed of lying or upright standing molecules

**Fig. 1:**
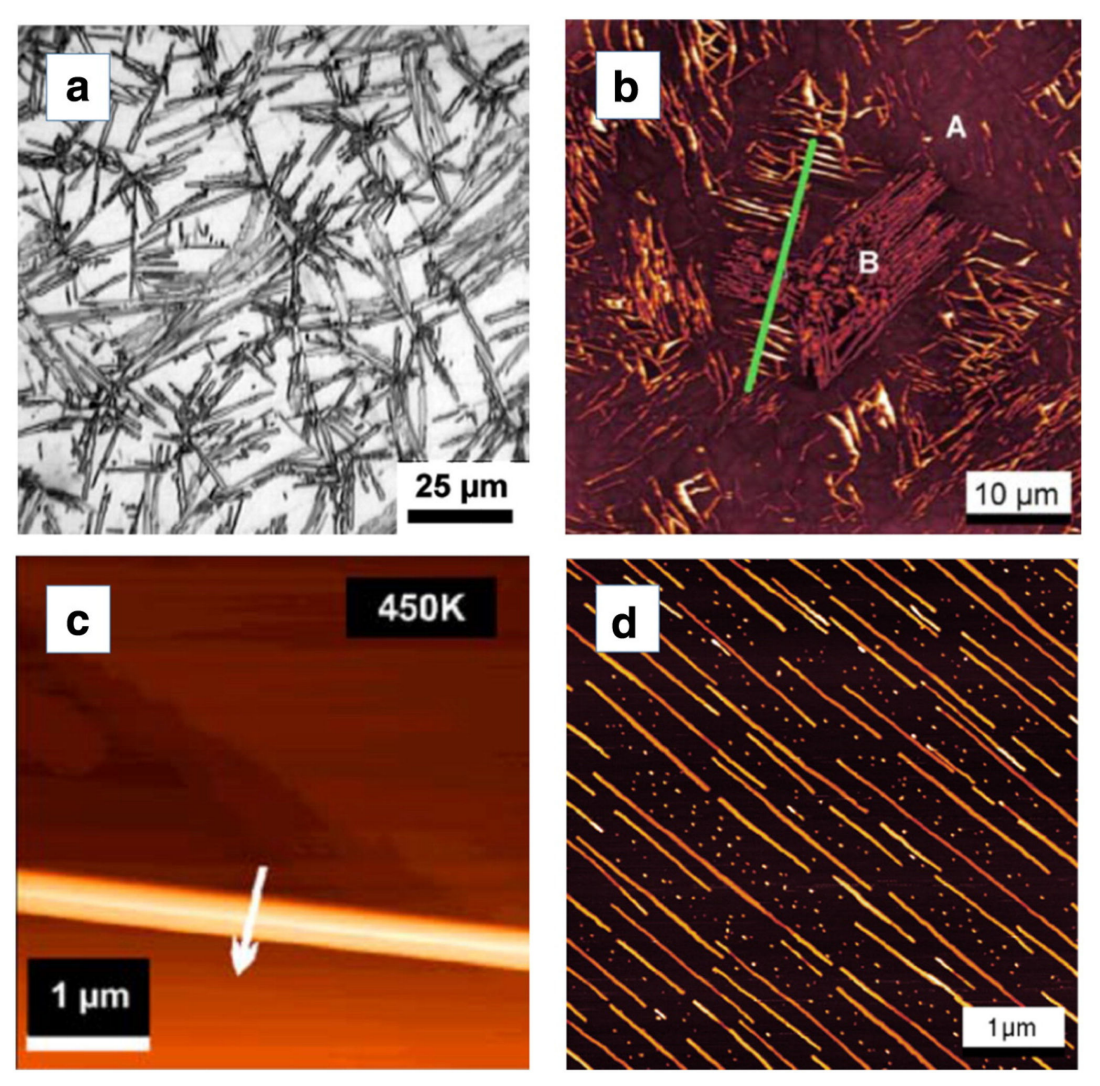
(a) OM image of 20 nm 4P on Au(111) at 300 K, (b) AFM image of 30 nm 6P on Au(111) at 430 K, (c) AFM image of 1 nm 6P on KCl(001) at 450 K, and (d) AFM image of 1 nm 6P on mica at 300 K. Reprinted with permission from Refs. [9] (a), [13] (b), [15] (c), and [4] (d).

When rod-like organic molecules are deposited on a substrate surface they will first adsorb in a flat-lying configuration, in which they diffuse along the surface and meet other molecules to form unstable or stable clusters, which further grow with increasing coverage and form islands. It turns out that basically two different island types can be observed, which are either composed of flat-lying molecules or of upright-standing molecules [6,7,8]. When the molecules are strongly bound to the substrate surface, not only the first monolayer is composed of lying molecules, but also the additional molecules in higher layers are incorporated in flat-lying orientation. Since both the diffusion probability as well as the incorporation probability are highly anisotropic, typically needle-like islands are formed in this case. Examples of such layer formation are 4P on Au(111) [9,10], 6P on Au(111) [11,12,13], 6P on KCl(001) [14,15] and 6P on crystalline mica [4,16,17]. In Fig. 1a an optical microscopy (OM) image is shown for a 20 nm thick 4P film deposited on Au(111) at room temperature, showing needle like crystallites of more than 20 μm length [9]. X-ray diffraction reveals that the crystallites are oriented with the 4P(211) plane parallel to the (111) plane of the gold surface. This plane is composed of molecules in a herringbone-like arrangement, where the long axes of the molecules are parallel to this plane and the short axes alternatively parallel or side-tilted. Moreover, XRD pole figures show that the molecules in the crystallites are aligned with respect to the arrangement of the gold atoms, either along the Au[110] or the Au[112] direction (epitaxial relationship) [10].

The growth of 6P films on Au(111) shows several similarities with that of 4P, however, a higher substrate temperature is needed to form long needle-like islands. While at 300 K only randomly oriented short crystallites with diameters around 100 nm are observed, films grown at 430 K exhibit needle-like crystallites of up to 10 μm length [13]. The reason for this difference is the smaller diffusivity of the longer oligophenylenes. A large-scale AFM image (Fig. 1b) shows that the needles are macroscopically oriented along distinct directions of the underlying substrate and rotated by 120°, again hinting at epitaxial growth. XRD and pole figure measurements reveal that in this case the 6P(213) plane is parallel to the Au(111) surface. The arrangement of the molecules in this plane is similar to that for the 4P(211) plane. The azimuthal orientation of the molecules is exclusively along the Au[110] direction [11].

6P deposited on KCl(001) exhibits a similar temperature dependent layer formation as described above. However, the diffusion probability at equivalent temperatures is much higher than on Au(111). While at and below room temperature only small crystallites form, the deposition at 450 K leads to extremely long needles (up to several 100 μm). The AFM image in Fig. 1c shows part of such a long needle which was formed after deposition of 6P at 450 K. The total amount of deposited 6P corresponds to just 1 nm mean film thickness [15]. Cross section measurements on this needle yield a width of about 200 nm and a needle height of about 150 nm. It needs to be mentioned that it was quite difficult to find the needles in the AFM at all, demonstrating the extremely high diffusion length of the 6P molecules on KCl at this temperature. Also noteworthy is the uniform width and height of these needles, which is probably caused by a stabilization due to strain within the needles. A detailed XRD investigation [18] showed that the long axes of the 6P molecules are again parallel to the (001) plane of the substrate, but the short axis is slightly side tilted, more precisely the 6P(203) plane is parallel to KCl(100). Furthermore, the long axes of the molecules are normal to the needle direction, which is along KCl[001].

A very special case is the film formation of 6P on muscovite mica (KAl_2_(AlSi_3_O_10_)(OH)_2_). Generally, well-defined mica substrates are produced by cleaving a mica sample along the basal plane, which produces an atomically flat crystalline surface. Cleavage proceeds along a potassium layer, which yields 0.5 monolayers of potassium in ionized form on each cleavage plane. Since in the lower lying silicon plane every fourth Si^4+^ ion is substituted by an Al^3+^ ion, the negative net charge in the subsurface layer and the same number of K^+^ ions on the surface form a dipole field with components parallel to the substrate [16]. This dipole field particularly causes the 6P molecules to be oriented parallel to the substrate, as shown for a 1 nm average thick film on mica, deposited at 330 K (Fig. 1d) [4]. It is remarkable that all needles are arranged in the same direction, indicating the strong influence of the dipole field. The needles can be up to 1 mm long. The needle length and the epitaxial order is again a function of the substrate temperature [19]. For the needle-like islands prepared around 330 K the 6P(111) plane is parallel to the substrate, whereas films prepared at 430 K show a strong fraction of crystals with the 6P(112) plane parallel to the substrate. In both cases the long axes of the 6P molecules slightly deviate from the orientation of the surface plane by about 4.7° [20].

A completely different growth behavior for rod-like molecules is observed, when the interaction strength between the molecules and the substrate is decreased. This can be either due to impurities on the surface, which typically reduce the surface energy, or due to surface amorphisation, which decreases the intimate contact of the carbon atoms within the organic molecules and the surface atoms, leading to decreased Van der Waals interactions. In this case, the molecule–molecule interaction becomes relatively more important, leading to islands composed of standing molecules, a configuration in which the interaction strength between the molecules and the substrate is reduced. To realize such a molecule arrangement the adsorbed flat-lying monomers have to reorient during nucleation and subsequent aggregation, which involves an activation barrier for this process. Therefore, also an increase of the surface temperature during film growth may additionally enhance the formation of islands composed of upright standing molecules. The regularly arranged molecules within the islands typically do not exhibit a commensurability with the arrangement of the surface atoms, thus no epitaxial growth is observed. Since under most experimental conditions the film growth is governed by the incorporation of diffusing monomers, the shape of the islands is more or less dendritic. Molecules, which adsorb and diffuse on top of an island cannot easily descend at the rim of the islands, due to an existing Ehrlich–Schwoebel barrier [21,22], thus the typical morphology of these films is that of terraced mounds. Very often, in particular on ill-defined surfaces, a mixture of needle-like islands composed of lying molecules and terraced mounds, composed of standing molecules, is observed [23].

**Fig. 2:**
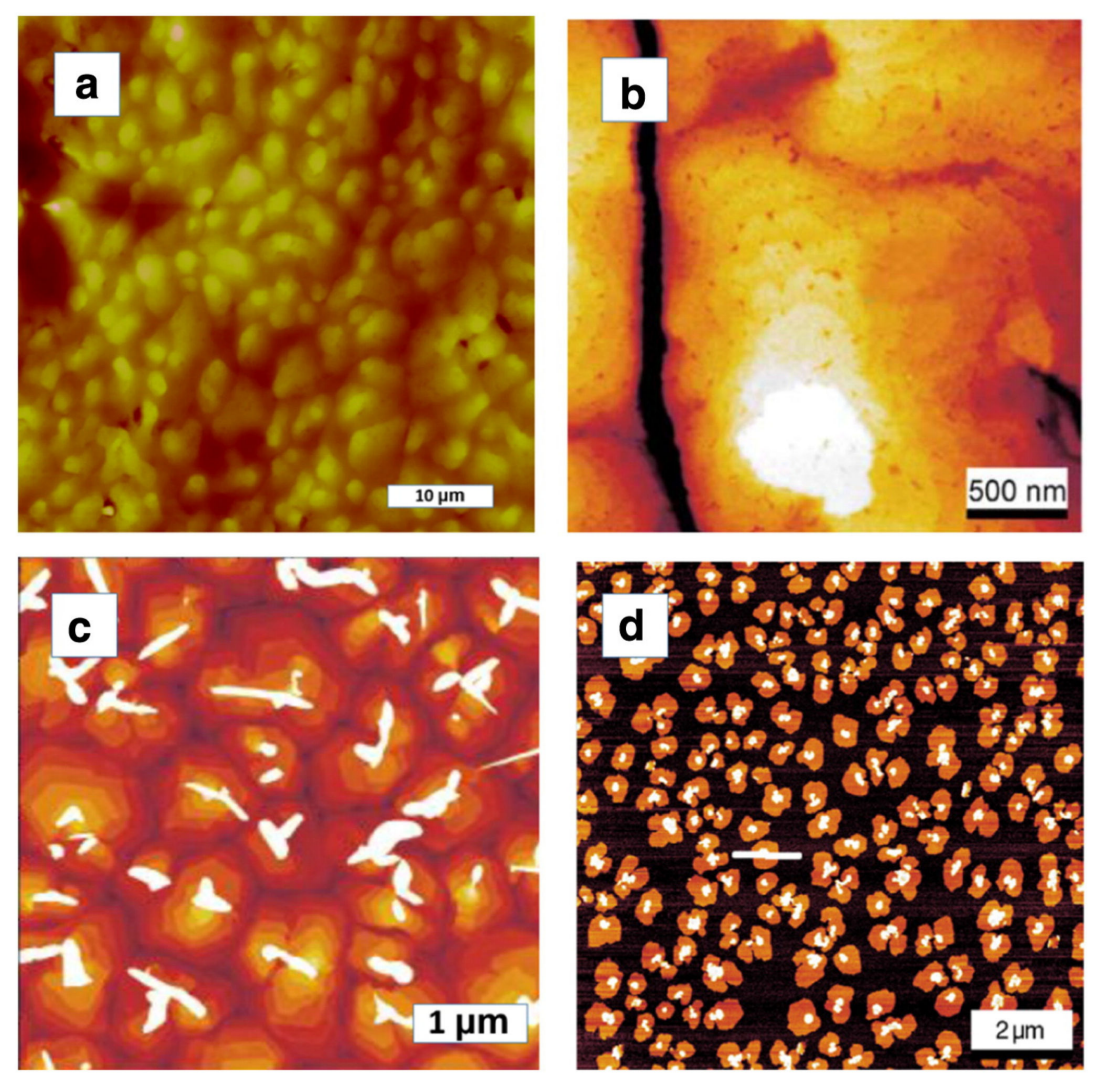
(a) AFM image of 30 nm4P deposited at 300 K on Au(111) + 0.5 ML carbon. (b) AFM image of 30 nm 6P deposited at 430 K on Au(111). (c) AFM image of 30 nm 6P on sputtered mica at 300 K. (d) AFM image of 1 nm 6P on C covered mica at 330 K. Reprinted with permission from Refs. [24] (a), [13] (b), [28] (c), and [4] (d).

In the following several examples of the latter type of islands are given: A 30 nm thick 4P film deposited at 300 K on a Au(111) surface, which was covered with just 0.5 monolayers of carbon, appears very homogeneous in optical microscopy, in contrast to that of Fig. 1a. However, AFM reveals that the film is composed of small, randomly oriented grains, with diameters of a few micrometer (Fig. 2a) [24]. XRD Ө/2Ө scans reveal that these islands are highly crystalline, with the 4P(001) plane being parallel to the gold surface. The orientation of the long axes of the 4P molecules is slightly tilted with respect to the normal of the (001) basal plane. No azimuthal alignment with respect to the Au(111) plane was observed [25]. In Fig. 2b one can nicely see the terraced mounds of 6P on Au(111), with step heights of 2.6 nm, which corresponds to the length of the 6P molecules. These islands were found between the needle-like islands on Au(111) after deposition at 430 K [13]. Indeed, on a carbon covered surface all the islands are composed of standing molecules, even down to a one monolayer thick film. This was demonstrated by LEED, which showed a ring-like pattern, indicating azimuthally randomly oriented domains with upright standing molecules [26,27]. The film growth on mica is a particular case, due to the dipole field on the mica surface, as described above. However, this dipole field can be disturbed either by the deposition of carbon or by sputter amorphisation. Fig. 2c shows the morphology of a 30 nm thick 6P film on sputtered mica, again exhibiting terraced mounds [28,29]. Interestingly, on top of these mounds needle-like crystallites nucleate. The reason for this behavior and the exact orientation of the molecules in these crystallites is not yet clear. A similar growth behavior can be seen for 6P on a 1 ML carbon covered mica surface [4]. For a 1 nm thick film all molecules are contained in slightly dendritic islands composed of standing molecules. Here, most molecules are contained in the first layer, but some have already nucleated in the second layer.

## 3 The existence of a wetting layer

**Fig. 3:**
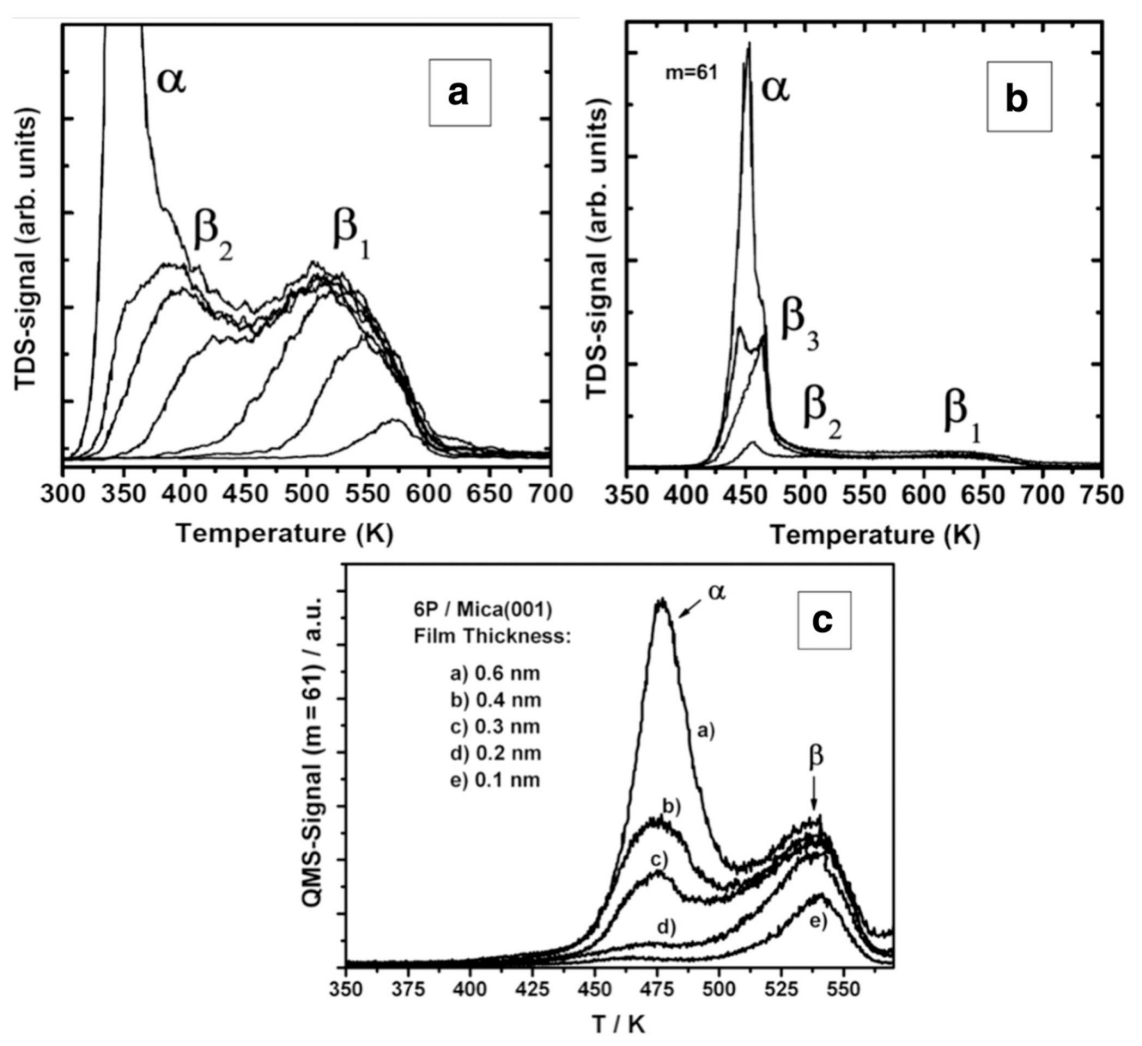
(a) TDS of 4P from Au(111). (b) TDS of 6P from Au(111). (c) TDS of 6P from mica(001). Reprinted with permission from Refs. [9] (a), [27] (b), and [4] (c).

One of the frequently discussed questions in the context of islandlike films relates to the existence of a strongly bound monolayer below and between the islands, a so-called wetting layer. A quite powerful method to identify the existence of a wetting layer is thermal desorption spectroscopy (TDS). Typically, the molecules in the wetting layer are more strongly bound to the substrate than the molecules within the overlying islands. This should lead to desorption peaks at different temperatures. A typical set of desorption spectra for such a scenario is shown in Fig. 3a for the system 4P on Au(111) [9]. For low coverage (up to 0.15 nm) 4P desorbs at around 550 K; this peak is labeled ß_1_. With increasing coverage a second peak (labeled ß_2_) appears at around 370 K, which saturates at about 0.3 nm. This coverage corresponds to a complete layer of flat-lying molecules. Detailed LEED investigations revealed that this monolayer is composed of 1/2 ML flat-lying (ß_1_-peak) and 1/2 ML side-tilted 4P molecules (ß_2_-peak) [10]. With increasing coverage a third desorption peak appears at about 350 K (labeled α-peak) which does not show saturation behavior. Thus, one can safely assume that this peak stems from desorption of molecules contained in the three-dimensional needle-like islands. The ß-peaks exhibit a shift of the peak maximum to lower temperature with increasing coverage, indicating a first-order desorption reaction with repulsive lateral interactions between the molecules in the wetting layer. Contrary, the asymmetric α-peak shifts to higher temperature with increasing coverage, a clear indication of zero-order desorption, representative for desorption from a 3D film [30,31]. The fact that the ß-peaks remain of the same size, independent of the size of the α-peak, clearly confirms the existence of a stable wetting layer of quaterphenyl below and between the needle-like islands.

A very similar desorption behavior is observed for 6P on Au(111) [27]. However, interestingly a third desorption peak (labeled ß_3_) which saturates at a coverage corresponding to 2 ML of lying molecules shows up before the multilayer α-peak appears, as shown in Fig. 3b. This indicates that in this particular case the wetting layer consists of two monolayers of flat-lying molecules. There exist many more examples for wetting layer behavior of rod-like organic molecules on metal substrates, e.g. 5A on Au(111) [32] and Ag(111) [33], 6P on Al(111) [34], but also on non-metallic substrates, e.g. 6P on TiO_2_(110) [35] or perfluoro-pentacene on silicon dioxide [36]. In all these cases the molecules are orientated parallel to the substrate, both in the monolayer as well as within the needle-like islands. A further example is the wetting behavior of 6P on mica(001), the corresponding TDS of which is displayed in Fig. 3c [4].

**Fig. 4:**
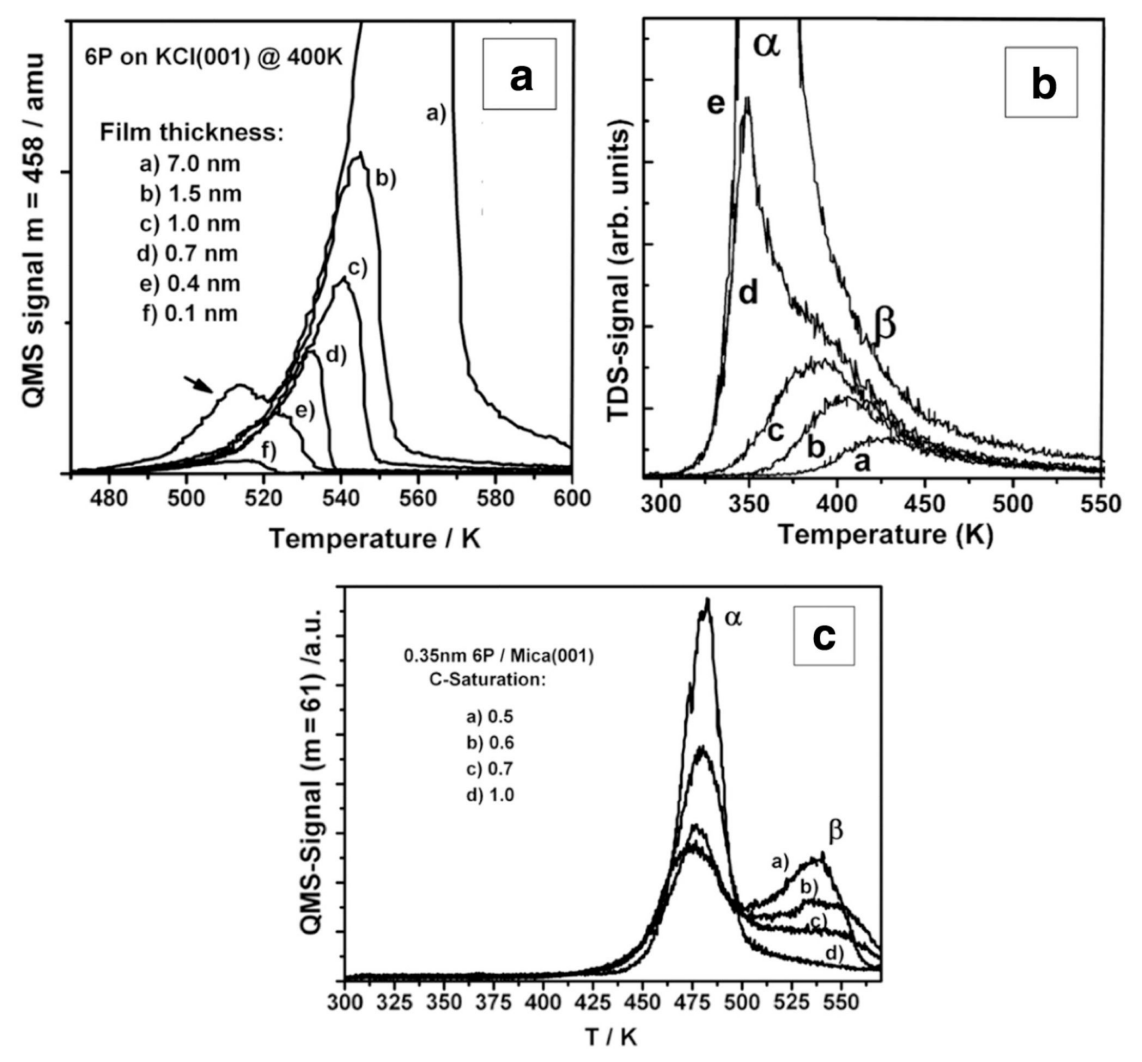
(a) TDS of 6P from KCl(001), showing no wetting layer (the peak marked by the arrow stems from the sample holder for high coverage). (b) TDS of 4P from Au(111), contaminated with 0.5 ML carbon. (c) Change of the wetting layer peak of 6P desorption from mica(001) substrates with increasing carbon contamination. Reprinted with permission from Refs. [14] (a), [38] (b), and [4] (c).

A different behavior can be observed for the system 6P on KCl(001) [37,14]. In this case, TDS provides proof that no wetting layer exists between the needle-like islands, and hence only a single peak appears in the desorption spectrum (Fig. 4a). Apparently, the bonding of the 6P molecules to the substrate is not strong enough to stabilize the wetting layer, but strong enough to maintain the molecules parallel to the substrate, even in the case of the 3D needle-like islands.

The question now arises to which extend surface contaminations influence the wetting layer. Several different scenarios can be observed, depending on the reactivity of the substrates and the extent of the contamination. For 4P on Au(111) the deposition of 0.15 ML of carbon changes the arrangement of the molecules in the wetting layer, as demonstrated by LEED [38], which has also consequences on the arrangement of the molecules in the multilayer. In addition to the 4P(211) planes also crystallites with the 4P(201) planes parallel to the Au(111) are observed by XRD [25]. In both cases, the molecules are still arranged with their long axes parallel to the substrate surface, the crystallites exhibit needle-like morphology. For a C-coverage of 0.5 ML a wetting layer (ß-peak) is still clearly visible in TDS (Fig. 4b), which saturates at 0.3 nm, meaning that the molecules are parallel to the surface, albeit no regular arrangement of the molecules in this wetting layer can be verified by LEED [38]. Further 4P deposition again yields the multilayer *α*-peak, which does not saturate. In this case, however, LEED and XRD investigations clearly show that the multilayer is composed of standing molecules. Correspondingly, the morphology of the film changed to plate-like islands [25]. The growth scenario for 6P on carbon covered Au(111) is similar to that for 4P. A wetting layer still exists on a surface covered with 2 ML of carbon, which saturates at about 0.3 nm, corresponding to one monolayer of flat-lying molecules. Again, LEED does not show a regular arrangement of the molecules within this wetting layer [26]. Further 6P deposition leads to islands in which the molecules are oriented in an upright position as it was already described above.

Finally, carbon contamination of the mica(001) surface plays a significant role for 6P growth. A series of TDS in Fig. 4c shows the decrease of the wetting layer desorption peak (ß) with increasing C coverage. At 1 ML of carbon, no wetting layer is present at all. Even at the smallest 6P coverage the molecules nucleate and aggregate into islands composed of standing molecules [4].

To summarize this chapter, four different growth scenarios were observed, depending on the molecules-substrate interaction strength: (a) Formation of a wetting layer of lying molecules with needle-like islands upon the wetting layer, also composed of lying molecules, (b) Formation of a wetting layer composed of lying molecules, with dendritic islands formed on the wetting layer, composed of standing molecules, (c) Formation of needle like islands composed of lying molecules directly on the surface, without a wetting layer, (d) Formation of dendritic islands composed of standing molecules, without a wetting layer. The growth scenarios of (a) and (b) are frequently ascribed in the literature as Stranski–Krastanov growth and that of (c) and (d) as Volmer–Weber growth. However, one should keep in mind that this classification, made originally by E. Bauer [39], refers to film growth under near-equilibrium conditions, whereas most of the films are grown far away from equilibrium and the morphology is kinetically limited.

## 4 The origin of bimodal island size distributions

**Fig. 5:**
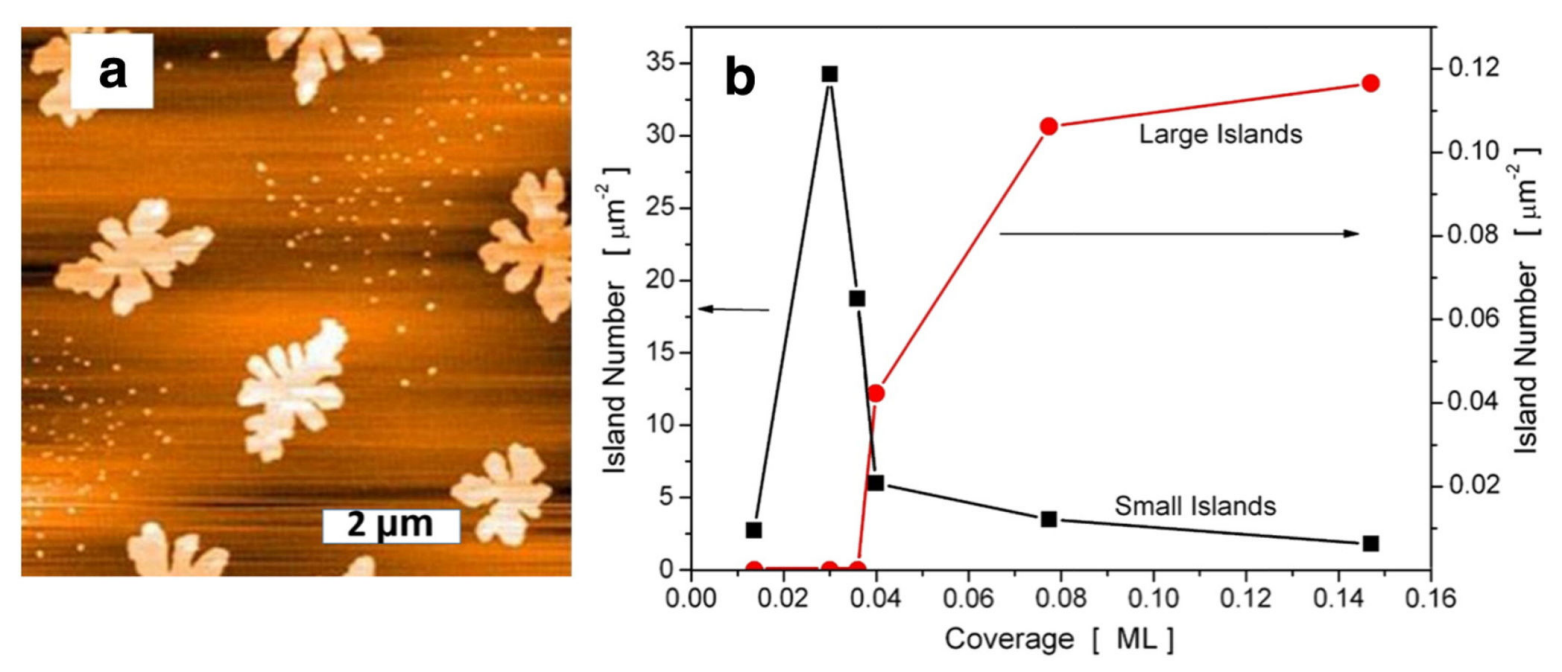
(a) AFM image of the bimodal island size distribution for 0.18 ML 6P on amorphised mica, deposited at 400 K. (b) Change of the number of small and large islands with increasing coverage of 6P on amorphised mica. Reprinted with permission from Ref. [41].

There exist several examples in the literature which show a quite unusual bimodal island size distribution for thin 6P films on mica. On crystalline mica(001) for example small compact islands can be observed between the needle-like islands, as shown in the AFM image of Fig. 1d [4]. Similar bimodal island size distributions have been observed for this system also by other research groups [17,19,40], however, a convincing explanation for this behavior has not been given at that time. Interestingly, for 6P on sputter amorphised mica, where the dendritic islands are composed of standing molecules, in a specific coverage range also a bimodal island size distribution can be observed, as shown in Fig. 5a. It could be experimentally verified that for both cases the small islands of the bimodal size distribution are due to subsequent nucleation, induced by venting the vacuum chamber for the ex-situ AFM investigations [41]. For the sputter-amorphised mica substrate, we have prepared a series of 6P films with different coverages, up to one monolayer of standing molecules (1 ML corresponds to a thickness of 2.6 nm). For very low coverages, up to 0.04 ML only small islands of standing molecules, with mean diameters of about 100 nm, can be seen in the AFM image. With increasing coverage suddenly much larger, dendritic islands with diameters of about 2 μm appear, which are surrounded by a region which is denuded of small islands. With further increase of the coverage, the number and size of the large islands increase, whereas due to increased denuded zones the total number of small islands decreases, as compiled in Fig. 5b. We interpret this behavior in the following way: At very small coverage a 2D gas phase of flat-lying monomers exists on the surface; the monomer density is not sufficient to nucleate stable islands of standing molecules under these conditions. However, when such a surface is exposed to air, increased diffusivity and/or a decreased activation energy for nucleation, most probably caused by the adsorption of water molecules, leads to venting-induced subsequent nucleation. The originating small islands are composed of standing molecules. We could simulate such a scenario by kinetic Monte Carlo calculations [41]. When the total coverage increases, some stable islands composed of standing molecules already form at the surface during deposition. These islands are in equilibrium with the monomer 2D gas phase. Venting of such a sample then leads to the bimodal island size distribution. The denuded zone around the larger islands may be viewed as a consequence of the decreased monomer density in the immediate vicinity of the stable islands, according to the diffusion-limited aggregation model [42]. At even higher coverages, above about 0.3 ML, most of the monomers are incorporated in the stable islands during deposition and the monomer density becomes exceedingly small. Hence, no small islands can form upon venting, leading to a single island size distribution. At even higher coverages (above 0.6 ML) coalescence sets in until finally a full monolayer develops. At this point, one should emphasize that indeed the first mono-layer nearly fully closes before islands in the second layer nucleate. Unfortunately, this is not the case for the additional layers, which leads to the well-known film morphology of terraced mounds. Apparently, the Ehrlich–Schwoebel barrier at the rim of the islands in the first layer is much smaller than for the higher layers [29,43].

**Fig. 6:**
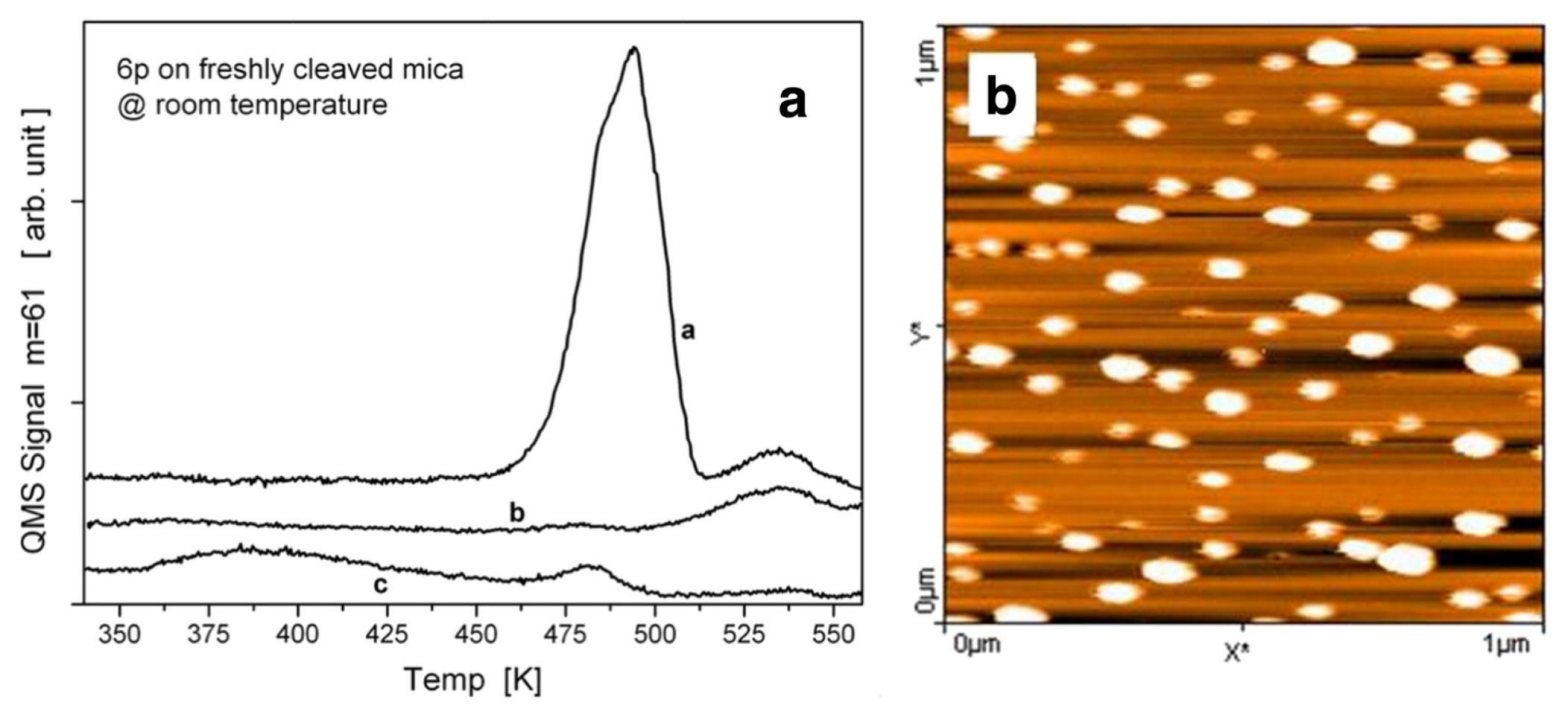
(a) TDS curves for 6P from crystalline mica with initial thickness of 1.6 nm (curve *a*) and 0.32 nm (curve *b*). Curve *c* shows the TDS after venting a sample with 0.32 nm 6P and reinstallation into the vacuum chamber. (b) Ex-situ AFM image of 0.32 nm 6P on crystalline mica. Reprinted with permission from Ref. [41].

The reason for a bimodal island size distribution on the crystalline mica(001) substrate, where needle-like islands coexist with small crystallites, is somewhat different. It is known that in this case a wetting layer exists, as demonstrated by TDS. Such a wetting layer could hardly be detected by AFM, anyway, we could show that this layer does not exist anymore after venting. In Fig. 6a several TDS traces are depicted. Curve *a* shows a spectrum after depositing 1.6 nm 6P on crystalline mica. In this spectrum, the first large desorption peak stems from needle-like islands and the second peak from the wetting layer, as already explained above. Curve *b* shows a spectrum obtained after depositing 0.32 nm 6P on the surface. Here, only the wetting layer exists, in agreement with the thickness of a full layer of flat-lying molecules of about 0.35 nm. One would expect that such a film does not show any structure in the AFM image. However, the opposite is the case, as shown in Fig. 6b. Many small crystallites cover the surface, which can only be explained by assuming again a venting induced dewetting and post-nucleation of the film. Actually, the evaluation of the AFM image with respect to the total coverage by integrating over all islands gives a mean thickness of 0.4 nm, in good agreement with the expected saturated wetting layer. The final proof for the venting induced dewetting yields a TDS of this sample when reinstalled into the vacuum chamber. In curve *c* in Fig. 6a the desorption peak representative for the wetting layer does not exist anymore. However, a peak as expected for desorption from 3D islands can clearly be seen. A broad additional peak at quite low temperature is most probably caused by coadsorbed water, as water can also be observed in the multiplexed mass spectrometer signal [41]. However, the total amount of desorbed 6P before and after venting is nearly the same. Thus, in a specific coverage range, when already during deposition needle-like islands have developed above the wetting layer, venting would again lead to the bimodal island size distribution due to subsequent dewetting of the wetting layer, including the denuded zones around the needle-like islands.

## 5 Attachment versus diffusion limited aggregation

One of the most relevant parameters in characterizing thin film growth is the critical island size *i*, essentially determining the morphology of the growing film. This quantity is defined as the largest not yet stable agglomeration of monomers. Incorporation of just another monomer leads to a stable cluster, which can further grow by aggregation. Clusters of monomers with size ≤ *i* can disintegrate again. The larger the critical island size the smaller is the number of stable islands and hence a coarse-grained film will develop and vice versa. Since the film morphology can have significant consequences on the physical properties of the film, the knowledge of the critical island size and the possible tailoring of this quantity is of great importance. For metal film growth at and below room temperature, the critical island size is commonly equal to 1, meaning that a dimer is already a stable cluster [44]. For rod-like organic molecules, however, there is ample evidence that this quantity is typically larger [5,45,46].

The most frequently applied model to describe island-like film growth is the diffusion-limited aggregation model (DLA) [42,47]. It implies that under usual experimental conditions the possibility that a monomer occupies an adsorption site by direct deposition from the gas phase is much smaller than that it is visited by a monomer diffusing on the surface in a given timeframe. In this model, it is assumed that a monomer, when it reaches another monomer or a stable or an unstable cluster, is incorporated with a probability of unity. Thus, the growth of the clusters is just limited by the diffusion flux, which in turn is determined by the deposition rate. At the beginning of film growth the number of stable islands increases, described by the nucleation rate, but above a specific coverage the number of islands reaches a maximum. In this regime, the so-called aggregation regime, no new islands can form, attributable to the monomer density becoming very small due to the immediate incorporation of the monomers into the islands [48]. Venables et al. [49] have demonstrated that the number of islands *N* in the aggregation regime is, inter alia, a function of the critical island size *i*:

(1) $$\frac{N}{N_{0}}=\eta (\Theta ,\;i){\left(\frac{4R}{\nu_{0}N_{0}}\right)}^{\frac{i}{i+2}}\exp\left(\frac{iE_{d}+E_{i}}{(i+2)\text{kT}}\right).$$

Here *N*_0_ is the number of adsorption sites, *R* the deposition rate, ν_0_ the attempt frequency for surface diffusion, *E_d_* the diffusion energy, *E_i_* the binding energy of the critical cluster, *k* the Boltzmann constant and *T* the surface temperature. *η* is a weak function of the coverage and *i*, with typical values in the range 0.2–0.3 [49]. According to this equation, the critical island size can simply be obtained by the deposition rate dependence of the island density in the aggregation regime. The slope *α* of the plot ln*N* vs ln*R* directly yields the critical island size, via *α* = *i*/(*i* + 2). Thus, the slope can only have values between 0.33 for *i* = 1 and 1 for *i* approaching infinity.

**Fig. 7:**
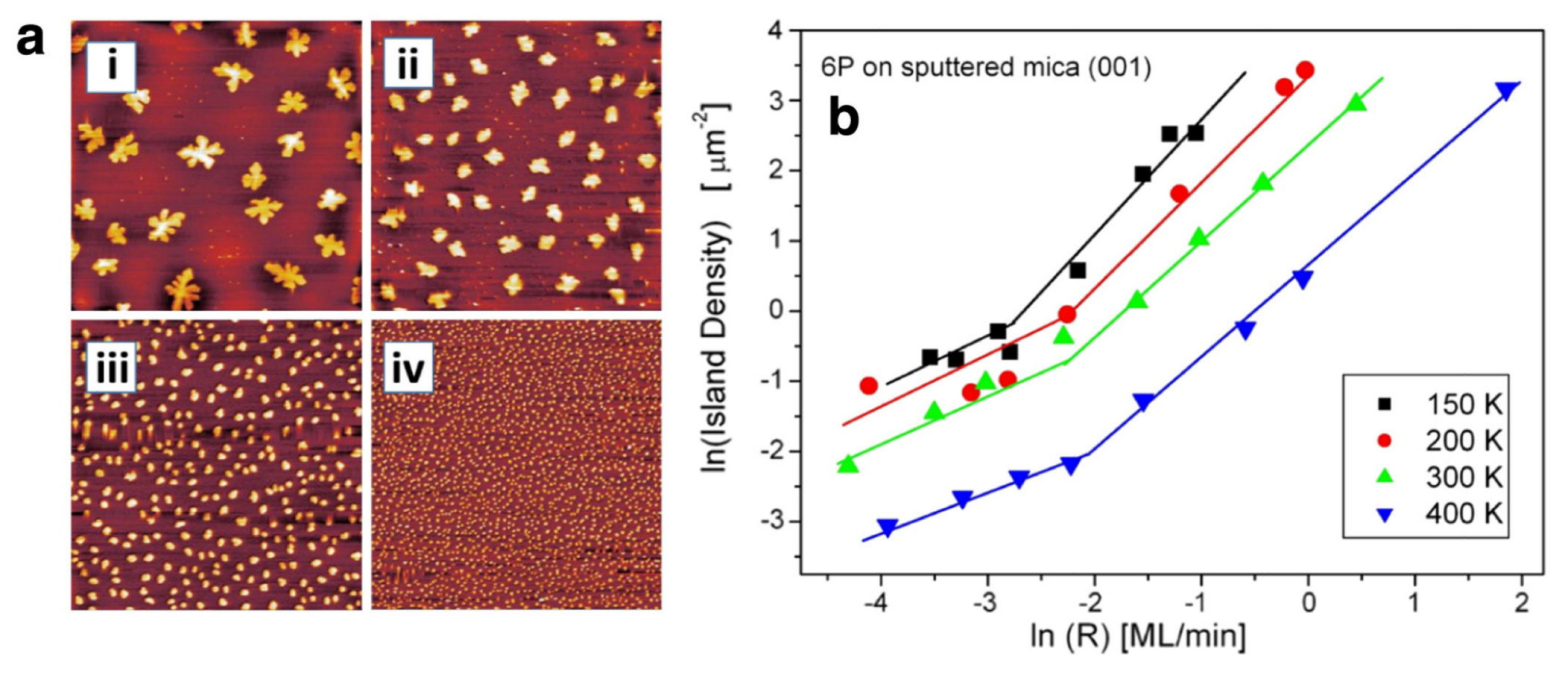
(a) AFM images (8 μm × 8 μm) of about 0.2 ML 6P deposited at amorphous mica at 200 K with different deposition rates: (i) 0.037 ML/min, (ii) 0.097 ML/min, (iii) 0.3 ML/min, and (iv) 0.8 ML/min. (b) Deposition rate dependence of the island density for 6P on amorphous mica, deposited at four different temperatures. Reprinted with permission from Ref. [50].

In this context, we have studied the nucleation and growth of 6P on sputter amorphised mica in detail [50]. In Fig. 7a a set of AFM images for 6P deposited at 200 K on amorphised mica is depicted for different deposition rates. One can impressively see the strong increase of the island density with increasing deposition rate. In Fig. 7b ln*N* vs ln*R* plots are compiled for different substrate temperatures. Evidently, there is not a single slope defining the critical island size, but for all temperatures a clear bend in the curves exist, indicating a cross-over between two different nucleation regimes. However, the most surprising result is that, whereas the slope at low deposition rate is about 0.7 ± 0.1, the slope increases to 1.4 ± 0.1 for high deposition rate. This is obviously incompatible with the DLA model. We proposed that the experimental result should be described by another aggregation model rather than by DLA, namely via attachment-limited aggregation (ALA). [50]. Indeed, such a scenario has been taken into account by Kandel [51], to describe the Si homoepitaxy on Si(111) with Sb as surfactant [52]. The main idea of the ALA model is that due to an activation barrier for the incorporation of the monomers at the rim of the islands (e.g. due to an adsorbed surfactant at the rim of the islands) the attachment probability is no longer equal to one, as assumed in the DLA model. One can clearly envision how such a scenario may also come into effect for the incorporation of rod-like organic molecules at the island rims. One needs to be aware that considerable reorientation of the flat-lying molecules will be necessary in order to be incorporated at the rim of an island consisting of standing molecules. By taking an activation barrier *E_b_* for attachment into account, Kandel [51], as well as Venables and Brune [53], derived a relationship between the island density and the deposition rate for such a scenario in the following form:

(2) $$\frac{N}{N_{0}}=\eta *(\Theta ,\;i){\left(\frac{4R}{v_{0}N_{0}}\right)}^{\frac{2i}{i+3}}\exp\left(\frac{2[i(E_{d}+E_{b})+E_{i}]}{(i+3)\text{kT}}\right).$$

While the general form of this relationship is similar to Eq. (1), the meaning of the exponent is changed to *α* = 2*i*/(*i* + 3). Thus, the slope can vary between 0.5 for *i* = 1 and 2 for large *i*, and the experimentally obtained value of *α* = 1.4 ± 0.1 is compatible with the ALA model. The quantitative evaluation of the data in Fig. 7b yields *i* = 5 ± 2 for low deposition rate, assuming DLA, and *i* = 7 ± 2 for high deposition rate, assuming ALA. A very similar result has also been found for the rod-like molecule pentacene deposited on amorphous mica [54], suggesting that the observed nucleation behavior is rather genuine for the film growth of rod-like organic molecules.

## 6 Hot-precursors involved in adsorption and nucleation

**Fig. 8:**
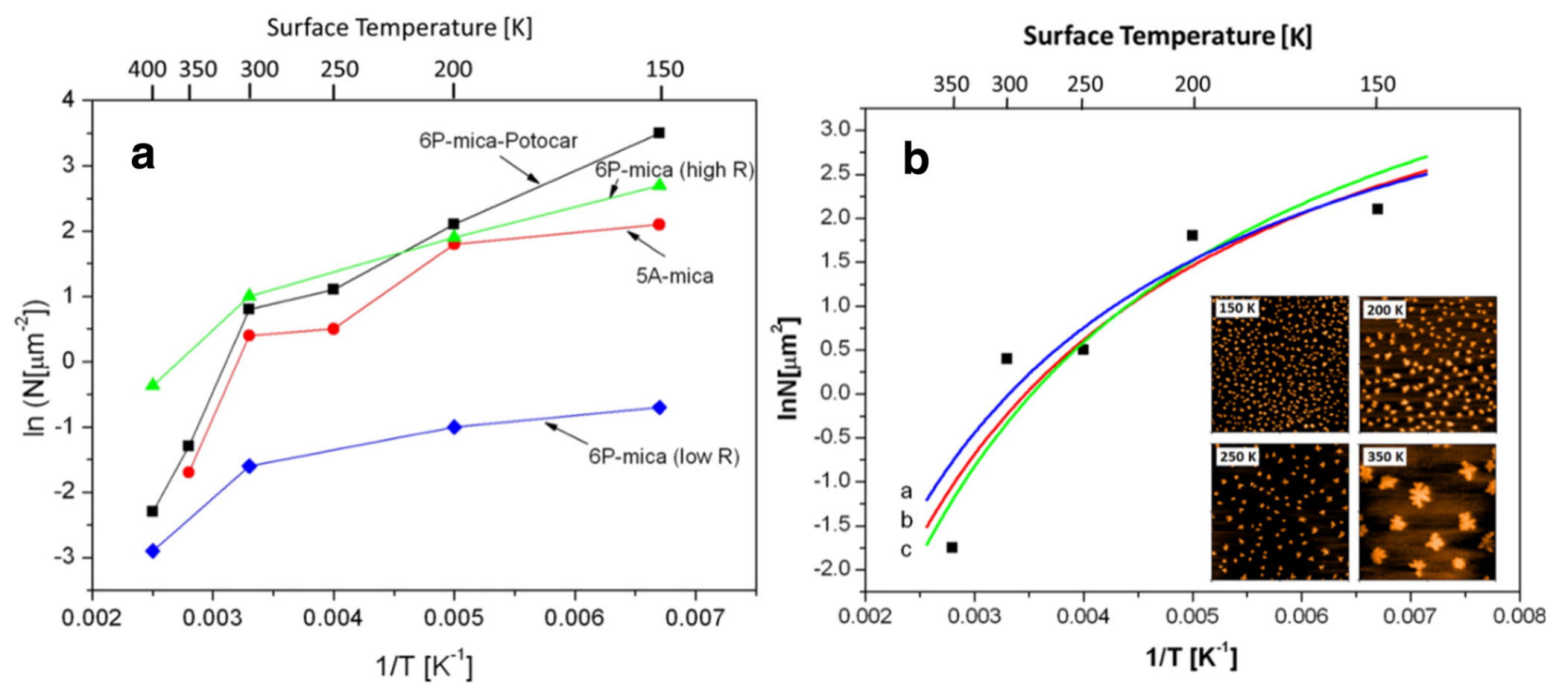
(a) Substrate temperature dependence of the island densities for various film/substrate systems, taken from Ref. [5] for 6P-mica-Potocar, from Ref. [50] for 6P-mica (high and low R) and from Ref. [58] for 5A-mica. (b) Experimental data points for 5A on mica, together with three fit curves, as described in the text. In the inset, some corresponding AFM images (8 μm × 8 μm) for different substrate temperatures are shown. Reprinted with permission from Ref. [58].

It is evident that the number of islands in the aggregation regime is basically determined by the experimental parameters of deposition rate and substrate temperature, in addition to system specific parameters. According to Eqs. (1) and (2), which describe this relationship for DLA and ALA, respectively, the island density ln*N* should be proportional to 1/*T*, for both cases. Thus, a plot of ln*N* vs 1/*T* should yield a straight line, whose slope is a function of the involved activation energies. Interestingly, a search in the literature reveals that in many cases a more or less strong deviation from a linear relationship is observed [55,56,57]. This is also true for the experiments carried out in our laboratory for 6P and 5A on amorphous mica. In Fig. 8a we compiled data for 6P on mica deduced from the work by Potocar et al. [5] and by Tumbek et al. [50], as well as for 5A on mica [58]. In spite of the large scatter of the data points a clear deviation from a linear relationship is observed. Furthermore, the slopes in the low temperature regime are way too small (in the order of 40 meV) to be physically reasonable for the involved energies *E_d_*, *E_i_* and *E_b_*.

In literature, different explanations have been given for the observed behavior. Berlanda et al. [56] proposed post-deposition nucleation and growth being responsible for the leveling off at low temperature. Ribič et al. [55] ascribed this phenomenon to the possible desorption at high temperature, while Yang et al. [57] made a change in the growth mechanism as a function of temperature responsible for the deviation of a straight line in the ln*N* vs 1/*T* plot. In our recent work [58] we proposed a quite different nucleation scenario that should account for the observed behavior. We suggest that the impinging molecules, which possess initially a kinetic energy according to the Knudsen cell temperature, cannot immediately dissipate their kinetic energy upon impact on the surface. Furthermore, excited rotational and vibrational states have to equilibrate. This will lead to a hot-precursor state in which the molecules are confined to the surface but have some transient mobility along the surface until they fully accommodate. Consequently, since the effective temperature of the molecules on the surface is larger than the surface temperature this leads to a smaller island density than calculated. The deviation increases with the difference between the evaporation temperature and the surface temperature. Although the existence and importance of transient mobilities in precursor states have frequently been questioned in the past, nowadays, this scenario is generally accepted. A comprehensive reference list to this subject can be found in a recent paper by Gao et al. [59].

It is quite difficult to describe the microscopic details of the formation and duration of a hot-precursor state and the subsequent processes which lead to nucleation. When an impinging molecule encounters the substrate surface, part of its initial kinetic energy and internal energy will be dissipated, but some part of the normal energy can also be converted into parallel kinetic energy and/or frustrated rotational motion. Furthermore, the molecule will be accelerated in the attractive potential and this energy can then also be partially converted into lateral motion. The rotational to lateral kinetic energy conversion will then depend on the orientation of the impinging molecule. When the molecule is finally trapped on the surface it will travel along the surface in a ballistic-like motion, where it can continuously lose energy by inelastic scattering with surface phonons until it fully equilibrates and any further motion can be described by random hopping. During this hyper-thermal sojourn the molecules can hit other molecules to form unstable or stable clusters, or become incorporated into an existing cluster. Clusters that are formed this way are not necessarily in equilibrium with the surface; hot molecules hitting the islands may transfer enough energy to break them apart or at least to detach single monomers. A comprehensive description of the processes for molecules in a hot-precursor and their contribution in aggregation would require detailed Molecular Dynamics and kinetic Monte Carlo simulations and has, to the best of our knowledge, not been performed on large organic molecules. However, a detailed analysis of hot-precursors based on rate equations has been performed recently by T. Einstein, A. Pimpinelli et al. [60,61].

For a semi-quantitative description of our experimental data we mimic the increased mobility in the hot-precursor state by a random diffusion of molecules with an effective temperature *T*_eff_, larger than the surface temperature [58]:

(3) $$T_{\text{eff}}(T_{\text{i}},T_{\text{s}},\kappa )=T_{\text{i}}-\kappa •(T_{\text{i}}—T_{\text{s}})$$

with *T*_i_ the temperature of the impinging molecules (i.e. the Knudsen cell temperature), *T*_s_ the surface temperature and *κ* being a coefficient which is related to the energy dissipation during the molecule impact at the surface and the sojourn in the hot-precursor.

In Fig. 8b three quantitative fits to the experimental data for 5A on mica are shown by applying Eqs. (2) and (3). Unfortunately, in addition to the accommodation coefficient *κ* also the involved energies and the frequency factor for diffusion *ν*_0_ are fit parameters. For simplification we assume a bond-breaking model for the binding energy *E_i_* ≈ (*i* — 1)*E_c_*, with *E_c_* being the binding energy between two molecules. Furthermore, we define a mixed energy *Ē* = *E_d_* + *E_b_* + *E_c_* [58]. The fit curves in Fig. 8b are derived with (a) *Ē* = 1.01 eV, *κ* = 0.23, (b) *Ē* = 0.99 eV, *κ* = 0.25, and (c) *Ē* = 0.98, *κ* = 0.27. The curvature of the fit is mainly determined by the coefficient *κ*. Only values between *κ* ≈ 0.15–0.3 lead to a proper curvature. For surface diffusion we have used the classical frequency factor of 10^–13^ s^–1^. However, since according to transition state theory, the rate constant *ν* is described by $\nu =\left(\frac{kT}{h}\right)\left(\frac{q^{‡}}{q}\right)$ with *q*^‡^ and *q* being the partition functions in the transition state and the adsorbed state [62], respectively, this value can be much larger for large organic molecules. This has not only been shown for desorption [31] but also for diffusion of organic molecules, e.g. *ν*_0_ = 2 × 10^17^ s^–1^ for 6P on sputtered mica [5]. Using such a diffusion frequency we obtain a best fit with *Ē* = 1.49 eV and *κ* = 0.19. Thus, in spite of the rather broad range of possible activation energies and frequency factors, we can set limits for the sum of the involved energies *Ē* ≈ 1–1.5 eV and the accommodation coefficient *κ* ≈ 0.19–0.25. The obtained values for the energies are much more realistic than those deduced by the classical evaluation, where diffusion of equilibrated molecules is assumed.

## 7 General scaling relationships for island film growth

The critical island size *i* can be experimentally obtained from the deposition rate dependence of the island density, as described above, according to *N ~ R^α^*. However, in order to derive the critical island size one has to know the exact physical mechanism for nucleation and aggregation [63]. In case of diffusion-limited aggregation (DLA) *α* = *i*/(*i* + 2) [53], whereas for attachment-limited aggregation (ALA) *α* = 2*i*/(*i* + 3) [51]. Additional evaluation methods have been proposed in the literature for the determination of *i*. It has been shown that also the island size distribution (ISD) for given *R* and *T* is determined by *i*. Amar and Familiy [64] have proposed an ad hoc analytical function for DLA, which has been frequently applied to determine *i* from experimentally obtained ISD [5,45,65,66]. More recently Pimpinelli and Einstein have proposed an alternative analytic approach to extract *i* based on the capture zone distribution (CZD) [67], which has been successfully applied for 6P growth on a variety of substrates [5,68]. Capture zones are approximated by Voronoi polygons, which are regions around the islands whose points are closer to the respective island center than to any other island center. The analytical form coincides with the so-called generalized Wigner distribution [69]:

(4) $$P({ß}_{s})=a_{ß}s^{ß}\exp\left(—b_{ß}s^{2}\right).$$

Here *s* = *v/V*, with *v* the Voronoi cell size, *V* the mean value of *v, a_ß_* and *b_ß_* are constants fixed by normalization and unit-mean conditions [67] and *ß* is a function of *i*. The most interesting aspect of the approach by Pimpinelli and Einstein is that the small area behavior of the CZD (determined by s^ß^) is dictated by the physics of the nucleation and aggregation process. It was conjectured that a proportionality exists between the probability of finding a given value of *s* and the probability of nucleating a new stable island [54]. The latter is proportional to the integral over the monomer density within the capture zone. The variation of the monomer density depends on the specific aggregation process. For instance, if the aggregation is diffusion limited, where the attachment is fast compared to diffusion, the monomer density vanishes at the island rim. On the other hand, for attachment limited aggregation the monomer density within the Voronoi cell remains rather uniform. Detailed calculations yield the result that the parameter *ß* in Eq. (4) is equal to *i* + 2 for DLA, but (*i* + 3)/2 for ALA [54].

**Fig. 9:**
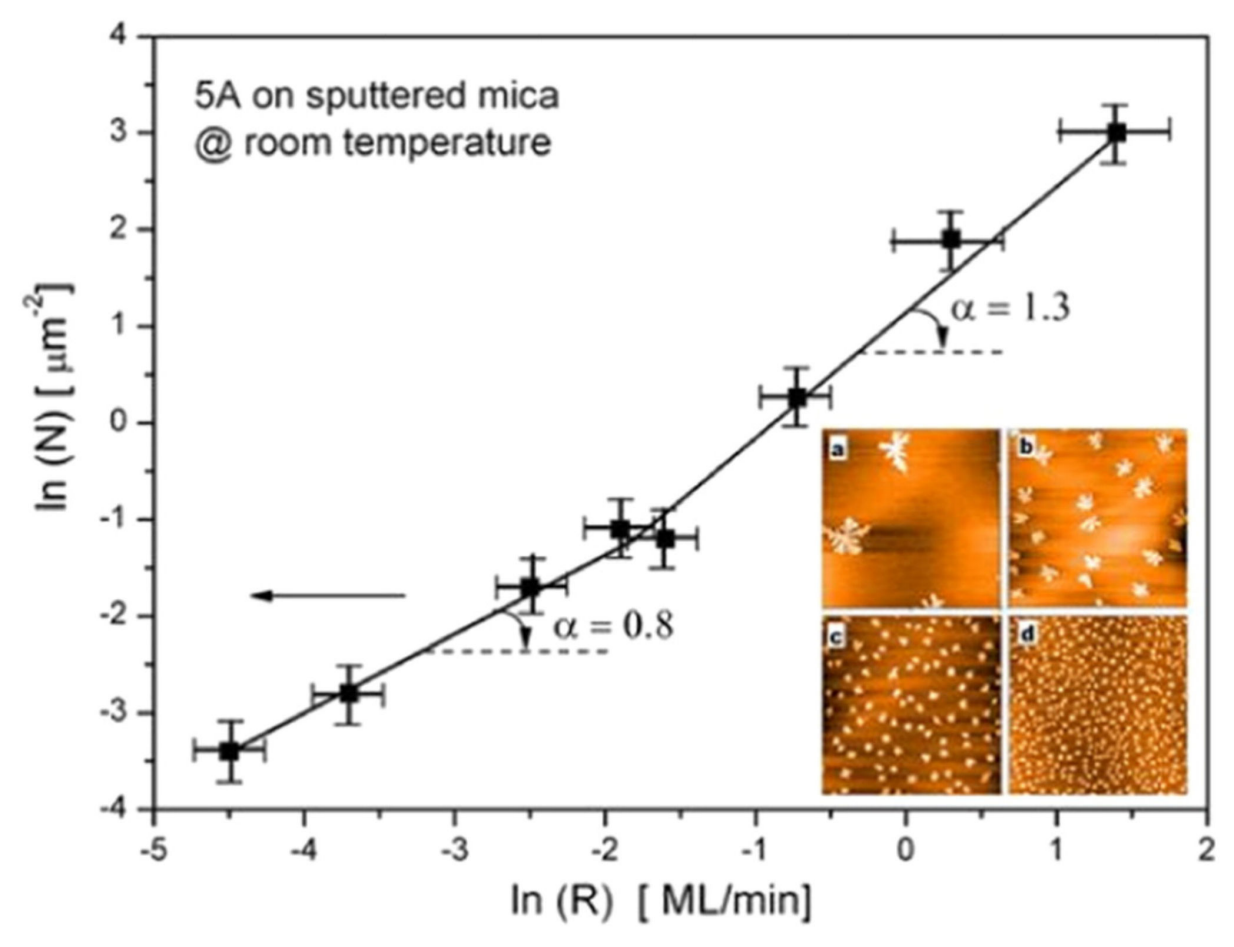
Island density of 5A on sputter amorphised mica as a function of the deposition rate at 300 K. The inset shows AFM images (8 μm × 8 μm) for different deposition rates. (a) 0.01 ML/min, (b) 0.15 ML/min, (c) 0.48 ML/min, and (d) 1.37 ML/min. Reprinted with permission from Ref. [54].

As a consequence of the considerations made above, the determination of the critical island size from CZD also requires the knowledge of the particular dominant aggregation process. In this context, we have studied the film growth of 5A on sputter amorphised mica. In Fig. 9 the deposition rate dependent island size distribution shows again a bend in the ln*N* vs ln*R* plot, with a slope of *α* = 0.8 ± 0.1 at low deposition rate, increasing to *α* = 1.3 ± 0.1 at high deposition rate [54]. This points to a similar cross-over from DLA to ALA, as observed for the system 6P on mica [50]. Evaluation of the critical island size *i* at low *R* (assuming DLA) yields a value of *i* with a large error, (5 < *i* < 18), for high *R* (assuming ALA) one obtains *i* ≈ 5.6 ± 1.

**Fig. 10:**
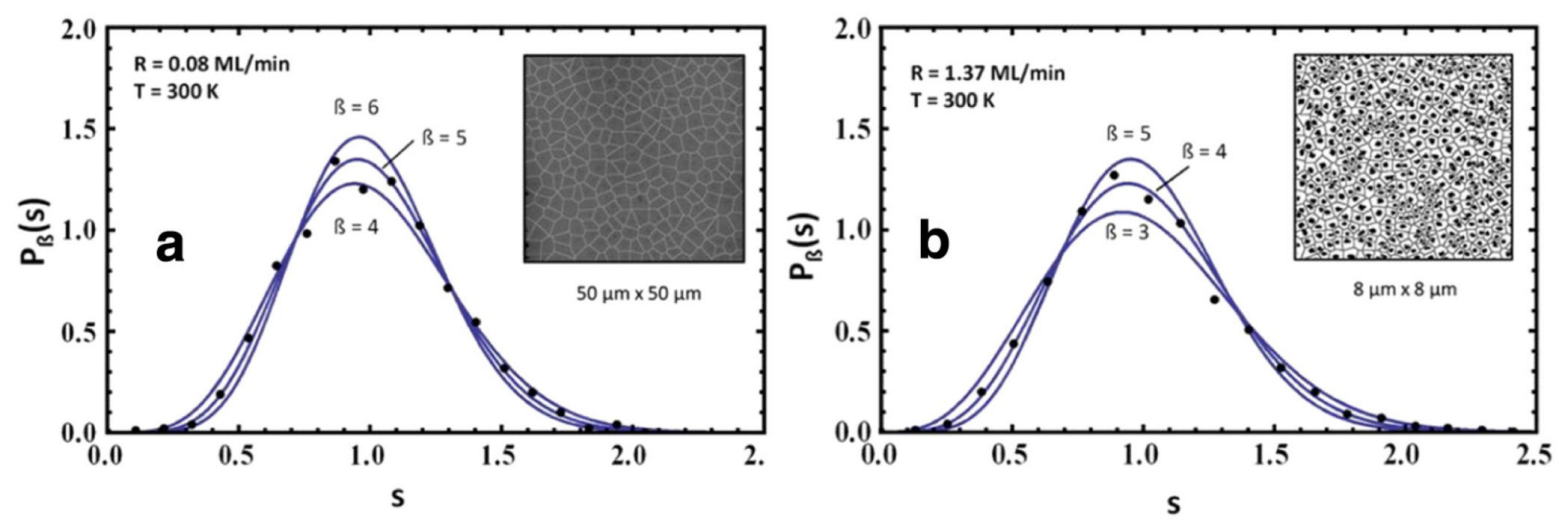
Capture zone distributions for 5A deposited on amorphous mica at 300 K with a deposition rate of (a) 0.08 ML/min and (b) 1.37 ML/min. The fit curves were calculated using Eq. (4). In the insets, representative Voronoi tessellations are shown. Reprinted with permission from Ref. [54].

Alternatively, we have measured the CZD for this system in the low and high *R* regime. This is shown in Fig. 10a,b. Fits are made to the experimentally obtained data points by using Eq. (4). Best fits are obtained with *ß* = 5 ± 1 for low *R* and *ß* = 4 ± 1 for high *R*. The deduced critical island sizes are *i* = 3 ± 1 for low *R* (assuming DLA) and *i* = 5 ± 2 for high *R* (assuming ALA). While the agreement for *i* between the rate dependent measurements and the CZD are very good for high *R*, they are less convincing for low *R*. This is most probably due to the fact that in the low *R* regime the aggregation cannot be described by a pure DLA mechanism.

One of the most impressive results of the considerations made by Pimpinelli et al. [54], however, is the simple, general relationship between the exponents *α* of the power law relation *N ~ R^α^* and the exponent *ß* in the CZD, *P*(*s*) ~ *s^ß^*, which reads

(5) α⋅β=i.

It turns out that this relationship should hold, independent of the specific aggregation mechanism. Thus, the independent measurement of *α* and *ß*, via the deposition rate dependent island density and the capture zone distribution, respectively, allows a rather precise determination of the critical island size *i*. Combining the data in Fig. 9 and Fig. 10 we obtain *i* = 4 ± 1 at low *R* and *i* = 5.2 ± 1 at high *R*.

## 8 Summary and conclusions

In the understanding of the initial steps of organic thin film growth, in particular for rod-like molecules, substantial progress has been made in the recent past. One is now in a position to make quite accurate predictions of the film morphology, energetics and kinetics as a function of the molecule/substrate system and of the preparation conditions, i.e. the deposition rate, substrate temperature and substrate conditioning. The film growth of oligo-phenylenes and acenes on metal and dielectric substrates can be considered as model systems, from which a number of conclusions can be drawn. In this short review article, I have focused on p-quaterphenyl, p-hexaphenyl and pentacene, deposited mainly on gold and mica substrates. One can roughly divide the substrates into rather reactive, in our case Au(111) and freshly cleaved mica(001), and rather unreactive, in our case carbon covered Au(111) and carbon covered or sputter-amorphised mica. On the reactive surfaces typically first a wetting layer of flat-lying molecules forms upon which islands grow, which are likewise composed of lying molecules. Due to the anisotropic diffusion and incorporation probability at the rim of the islands, these islands have a needle-like shape (e.g. 4P-Au(111) [9], 6P-Au(111) [13], 6P-mica(001) [4]). On the rather unreactive surfaces, typically no wetting layer forms and the molecules nucleate into islands, composed of upright-standing molecules. These islands have a more or less dendritic shape, due to diffusion-limited aggregation (e.g. 6P-mica + C [4], 6P sputtered mica [4], 5A-sputtered mica [54]). In some cases a wetting layer of lying molecules forms, on which dendritic islands composed of standing molecules grow (e.g. 4P-Au(111) + C [38], 6P Au(111) + C[26]).

A caveat in the interpretation of the film morphology, typically measured by ex-situ AFM, and the comparison with in-situ measured film properties, is the occurrence of venting-induced nucleation. This has been shown to take place for 6P and 5A on crystalline and sputter-amorphised mica [41]. In particular very thin layers, e.g. wetting layers, may dewet and form islands due to the exposure to air, most probably due to water coadsorption. In a particular coverage regime, when during deposition already nucleated islands are in equilibrium with the 2D gas phase, venting induced subsequent nucleation leads to a pronounced bimodal island size distribution. Thicker films are of course less prone to subsequent dewetting.

A quite important issue in the film growth of extended organic molecules is the question, whether the nucleation can be described by the well-known models derived for point-like monomers, e.g. for metal atoms. The latter is successfully described by the diffusion-limited aggregation (DLA) model, in which it is assumed that a critical island size has to be surmounted to get stable islands. Furthermore, it is assumed that the incorporation probability of the diffusing monomers at the rim of the islands is unity. We could show that the latter assumption is no longer valid in the case of rod-like molecules [50]. It is easy to understand that for the flat-lying elongated monomers, when they encounter the rim of an island composed of standing molecules, considerable reorientation has to take place, leading to an activation barrier for attachment. Experiments on the deposition rate dependence of the island density in the aggregation regime have shown that the classical nucleation model derived by Venables et al. [49] for DLA, cannot explain the results. However, an attachment-limited aggregation (ALA) model, proposed by Kandel [51], allows a satisfactory description of the experiments. A critical island size of about 5 pentacene molecules and of about 7 hexaphenyl molecules on amorphous mica was obtained from the deposition rate dependence of the island density.

Within the nucleation models, either for DLA or for ALA, it is assumed that the impinging molecules immediately accommodate to the surface temperature and diffuse on the surface in a random walk like motion. However, we could unequivocally demonstrate that this assumption does not allow the description of the temperature dependence of the island density properly [58]. In fact, the impinging molecules, which possess kinetic energy according to the temperature of the evaporation cell, will be trapped in a so-called hot-precursor state, in which the molecules can move along the surface for some time in a ballistic-like motion, until they finally become thermalized due to sufficient phonon excitations. The consequence of this hyper-thermal diffusion is that less, but larger islands nucleate than under thermal equilibrium conditions. This effect increases with the difference between the evaporation cell temperature and the surface temperature.

The above-mentioned critical island size *i* is an important parameter, because it determines the grain size of the film. This information is important for many applications of the organic film. One method to get information on this quantity is the above-mentioned relationship between the island density *N* and the deposition rate *R*. The relationship has the form *N ~ R*^*α*(*i*)^, with *α* = i/(*i* + 2) for DLA [49] and *α* = 2i/ (*i* + 3) for ALA [51]. It was suggested in literature that the island size distribution (ISD) [64] and/or the capture-zone distribution (CZD) [67] can also be used to extract information on the critical island size. In particular, for the CZD Pimpinelli and coworkers [54] and [67] have proposed an analytic relationship in the form *P*(*s*) ~ *s*^*ß*(*i*)^. Here, *s* is the size of the capture zones, *P*(*s*) the probability of capture-zones with the size *s* and the fit parameter *ß* is a function of *i*, with *ß* = *i* + 2 for DLA and *ß* = (*i* + 3)/2 for ALA. The disadvantage for the application of the presented relationships is that the exact aggregation scenario, either DLA or ALA, has to be known. However, the most impressive result of the considerations by Pimpinelli et al. [54] is that the relationship α·ß = *i* holds, independent of the specific aggregation scenario. Thus, the independent determination of α from the deposition rate dependence of the island density, and *ß* from the capture zone distribution, allows the explicit determination of the critical island size.
